# A deep learning based method for left ventricular strain measurements: repeatability and accuracy compared to experienced echocardiographers

**DOI:** 10.1186/s12880-024-01470-7

**Published:** 2024-11-12

**Authors:** Magnus Rogstadkjernet, Sigurd Z. Zha, Lars G. Klæboe, Camilla K. Larsen, John M. Aalen, Esther Scheirlynck, Bjørn-Jostein Singstad, Steven Droogmans, Bernard Cosyns, Otto A. Smiseth, Kristina H. Haugaa, Thor Edvardsen, Eigil Samset, Pål H. Brekke

**Affiliations:** 1 Institute for Clinical Medicine, University of Oslo, Oslo, Norway; 2 ProCardio Center for Innovation, Department of Cardiology, Oslo University Hospital, Rikshospitalet, Oslo Norway; 3 Institute for Surgical Research, Oslo University Hospital and University of Oslo, Oslo, Norway; 4 Department of Cardiology, Oslo University Hospital, Rikshospitalet, Oslo Norway; 5 Centrum Voor Hart-en Vaatziekten, Universitair Ziekenhuis Brussel-Vrije Universiteit Brussel, Brussels, Belgium; 6 Department of Cardiology, Universitair Ziekenhuis Brussel, Jette, Belgium; 7 Centrum Voor Hart- en Vaatziekten, Universitair Ziekenhuis Brussel, Brussels, Belgium; 8 Institute of Surgical Research, Oslo University Hospital, Rikshospitalet, Oslo, Norway; 9 Faculty of Medicine, University of Oslo, Oslo, Norway; 10 Faculty of Medicine, Karolinska Institutet and Cardiovascular Division, Karolinska University Hospital, Stockholm, Sweden; 11 GE Healthcare, Oslo, Norway; 12 Department of Informatic, University of Oslo, Oslo, Norway

**Keywords:** Speckle-tracking echocardiography, Strain rate imaging, Deep learning, Artificial intelligence, Automation

## Abstract

**Background:**

Speckle tracking echocardiography (STE) provides quantification of left ventricular (LV) deformation and is useful in the assessment of LV function. STE is increasingly being used clinically, and every effort to simplify and standardize STE is important. Manual outlining of regions of interest (ROIs) is labor intensive and may influence assessment of strain values.

**Purpose:**

We hypothesized that a deep learning (DL) model, trained on clinical echocardiographic exams, can be combined with a readily available echocardiographic analysis software, to automate strain calculation with comparable fidelity to trained cardiologists.

**Methods:**

Data consisted of still frame echocardiographic images with cardiologist-defined ROIs from 672 clinical echocardiographic exams from a university hospital outpatient clinic. Exams included patients with ischemic heart disease, heart failure, valvular disease, and conduction abnormalities, and some healthy subjects. An EfficientNetB1-based architecture was employed, and different techniques and properties including data set size, data quality, augmentations, and transfer learning were evaluated. DL predicted ROIs were reintroduced into commercially available echocardiographic analysis software to automatically calculate strain values.

**Results:**

DL-automated strain calculations had an average absolute difference of 0.75 (95% CI 0.58–0.92) for global longitudinal strain (GLS), and 1.16 (95% CI 1.03–1.29) for single-projection longitudinal strain (LS), compared to operators. A Bland–Altman plot revealed no obvious bias, though there were fewer outliers in the lower average LS ranges. Techniques and data properties yielded no significant increase/decrease in performance.

**Conclusion:**

The study demonstrates that DL-assisted, automated strain measurements are feasible, and provide results within interobserver variation. Employing DL in echocardiographic analyses could further facilitate adoption of STE parameters in clinical practice and research, and improve reproducibility.

## Introduction

Global longitudinal strain (GLS) using speckle tracking echocardiography (STE) is a validated and robust measurement for left ventricular (LV) function. GLS is reported to be a sensitive marker for LV systolic function, and provides incremental prognostic and diagnostic value in coronary heart diseases [1], early systolic dysfunction [2, 3], cardiomyopathies and valvular disease [4–8]. While STE parameters are widely used in research settings, use in clinical practice is more limited, partly due to the labor and experience required to manually delineate myocardial walls for strain analysis.

Outlining structures or objects in images, i.e. segmentation, using deep learning (DL) has shown great potential in fields such as ophthalmology [9], skin cancer [10] and radiology [11]. Automating echocardiographic analysis using artificial intelligence (AI) technology, such as neural networks, has the potential to reduce operator-dependent variability and analysis time, while increasing repeatability. Studies have already demonstrated the feasibility of fully automated GLS calculations, including both view recognition and analysis [12, 13], and the use of commercial software for automated strain calculations [14, 15].

As a rule of thumb, the quality of a DL model is dependent on the size of the training data set and the accuracy of its labeling (in AI parlance: “ground truth”). In medical imaging, and echocardiography in particular, access to large datasets is limited. Also, with the low signal-to-noise ratio of ultrasound images, and the echocardiographic inter- and intraobserver variability, questions arise regarding which aspects of DL training matter most: data quantity, ground truth quality, or even transfer learning from other data sets.

With increasing usage of DL models in all aspects of life, the sometimes extraordinary failures [16, 17] of such tools in seemingly ordinary situations have become popular memes. While a few mistakes may not have a large impact on model statistics, this “black box” problem [18] could be significant in a clinical setting if the operator does not understand what is happening or is unable to intervene. Indeed, the American Society of Echocardiography (ASE)/ European Association of Cardiovascular Imaging (EACVI) guidelines recommend that all medical imaging diagnostics using speckle tracking and automatic segmentation must allow the operator to visually check the tracking results and to manually correct them to account for mislabeling and varying anatomy [19].

In the current study, we aimed to integrate a DL-based automatic method for LV segmentation, trained on a large, clinical echocardiographic dataset, with commercially available echocardiographic analysis software. Thereby retaining the clinical workflow with a human in the loop, and the possibility for an operator to inspect and correct every measurement, while reducing analysis time. Employing commercial software in use world-wide for strain calculations means GLS results from the present study are directly comparable to clinical data. Furthermore, we aimed to investigate how transfer learning, data quantity, and data quality affect the DL-assisted GLS calculations, in addition to validating DL models built on the open source CAMUS echocardiographic data set [20] on clinical strain measurements from our hospital. The study aims to be a proof of concept, focusing on testing the feasibility and basic functionality of the idea, not to develop a fully operational pipeline ready for deployment.

## Methods

### Study population

Echocardiographic exams were collected from available datasets used in earlier research projects by our group (Center for Cardiological Innovation/ ProCardio Center for Innovation) between 2006 and 2018, and all available STE echocardiograms acquired related to invasive coronary angiography performed at Oslo University Hospital Rikshospitalet in 2018. The dataset consisted of 672 echocardiographic exams from 605 patients, acquired at Oslo University Hospital Rikshospitalet and University Hospital Brussels. Age was 63.4 ± 17.5 years, gender distribution 61.5% male. This included examinations from patients with aortic stenosis (*n* = 121), Brugada syndrome (*n* = 111), Mitral valve prolapse (*n* = 22) hypertrophic cardiomyopathy (*n* = 54), patients with heart failure before and after cardiac resynchronization therapy device implantation (n_before_ = 72, n_after_ = 67), and patients with myocardial infarction (*n* = 219). There were also a small number of examinations from patients with no known heart disease (*n* = 6). 453 (67%) examinations were acquired for research projects, while 219 (33%) were clinical exams. All data were anonymized upon extraction, leaving only age, gender, and primary diagnosis. Using stratified randomization based on diagnosis, the examinations were divided into three sets, with 15% of data reserved for testing of clinical measurements while the remaining 85% was split into training and validation sets (Table 1). The test set consisted of 307 images from 107 patients, with all 3 apical views present in 83 (76%) patients.

**Table 1:** Stratifiction of diagnosis, quality and acquisition setting in training and validation set, and test set

	**Training and validation set**					**Test set**
**Subset**	**K1**	**K2**	**K3**	**K4**	**K5**	
Total number of projections	331	331	331	322	312	307
**Diagnosis**						
Aorta stenosis	59	64	62	57	58	50
Mitral valve prolapse	12	11	12	11	9	8
Brugada syndrome	57	57	57	62	52	52
Hypertrophic cardiomyopathy	28	22	21	26	27	28
Myocardial infarction	98	93	100	89	89	88
Heart failure	72	80	75	73	74	75
Healthy	5	4	4	4	3	6
**Quality**						
High	17	30	24	32	21	33
Medium	206	182	212	198	200	183
Low	108	119	95	92	91	91
**Image acquisition setting**						
Clinical	126	115	121	115	116	116
Research	205	216	210	207	196	191

Two open source datasets were employed for transfer learning [21] and external validation: ImageNet ILSVRC is a commonly used open source database with thousands of images, and is often used for benchmarking segmentation models [22]. The CAMUS dataset is a publicly available echocardiographic dataset consisting of 500 patients with annotated epicardial and endocardial border [20].

The echocardiographic examinations originated from Vivid E9 and E95 ultrasound systems (GE Healthcare, Horten, Norway). Clinical image analyses were performed using EchoPAC software version 201, 202, and 203 (GE Vingmed Ultrasound). The echocardiograms were primarily acquired and analyzed by trained cardiologists following the EACVI/ASE clinical recommendations, and then quality assessed by a second cardiologist with 20 years of echocardiographic experience.

### Data pipeline and model development

Mid-systolic frames and corresponding LV region of interest (ROI)s were extracted from image loops using GE proprietary software and exported for analysis on an offline workstation. The extracted images, and the ROI masks, were in 8-bit grayscale, 256 × 256 pixels. All images were manually reviewed to eliminate single wall-, right ventricle-, and left atrial strain exams from the data set. The quality of each image, and the placement of the corresponding mask, were quality assessed by an experienced cardiologist and determined to be either of low, medium, or high quality based on image noise and contrast, endo- and epicardial border visibility, and accuracy of LV outline markers.

**Fig. 1:**
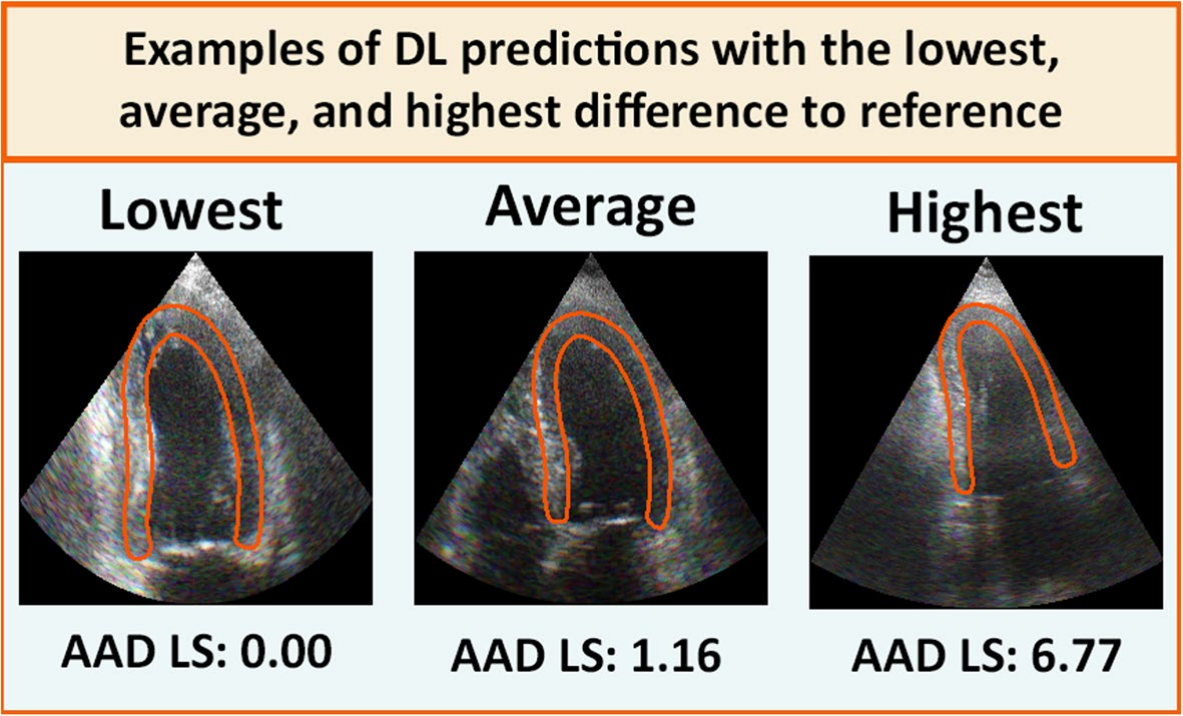
Examples of DL predicted ROI overlaid on corresponding echocardiographic image AAD = Average absolute difference, LS = Single projection longitudinal strain

In the current study, convolutional neural networks (CNNs) were trained in a supervised way [23]. The model was provided with examples of echocardiograms and the corresponding ROI mask, and the model would then try to learn the relationship between these. A successfully trained model will be able to output a ROI mask for any given echocardiogram (Fig. 1).

5-fold cross-validation [24] was applied on the train/validation data during development of the model in order to estimate the model's performance and select the right model and parameters. EfficientNetB1 [25] was chosen as encoder, as it was the state-of-the-art CNN architecture based on the benchmarking dataset ImageNet at the time of choosing (September.2020), and allows for easy implementation of transfer learning. Furthermore, we used a U-net based encoder, and ADAM as the optimizer. As for the loss function, a combination of Dice score and Binary Cross Entropy was determined to be the most consistent. The model was trained for 30 epochs with a batch size of 20 and a learning rate of 0.001. The code used for training is available at https://github.com/shigurd/DL_ECHO/tree/ed9053926f0a520c8271f53f87db5d26019eee9b/LV_segmentation.

Image augmentation was employed to increase variation in the data set. Employed augmentations included rotation, shifting, zooming, horizontal and vertical warping, adding gaussian noise and gamma adjustments, all within clinical plausibility. The augmentations were chosen randomly, with multiple augmentations being done on each image. The code used for augmentations is available at https://github.com/shigurd/DL_ECHO/blob/ed9053926f0a520c8271f53f87db5d26019eee9b/data_partition_utils/create_augmentation_imgs_and_masks.py, and includes augmentation ranges for all utilized augmentations.

Finally, the trained model was used to generate ROIs from echocardiograms, and these ROIs were then reintroduced into EchoPAC version 203 using a custom script. EchoPAC was then used to calculate LS and GLS following standard clinical procedure. GLS was calculated for all patients where all three apical views were available.

### Data quality and network property testing

Data set properties effect on model performance were assessed by training two separate models, one on all data, and one restricted to high and medium quality. However, there was insufficient high-quality data available to train a separate model only on high quality data. Separate models were also trained using data acquired in either a research or clinical setting. To evaluate the effect of dataset size, separate models were trained starting with 100 patients, and increasing by 100 patients every step until all data was included.

**Fig. 2:**
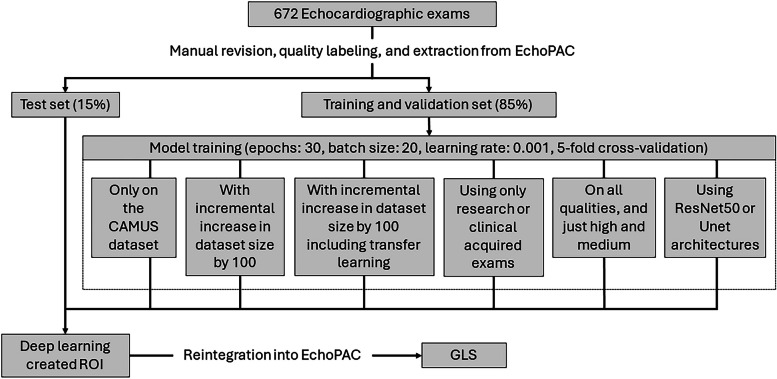
Workflow of data pipeline and model training GLS = Global longitudinal strain, ROI = Region of interest

We studied the impact of transfer learning by initializing models using weights from previous models trained on either ImageNet or the CAMUS dataset. Additionally, U-net [26] and ResNet50 [27] encoder architectures were tested using the highest scoring techniques and parameters previously mentioned. An overview of tested parameters can be found in Fig. 2.

### CAMUS validation

Finally, a model trained on the publicly available CAMUS dataset, using the optimal architecture and settings discovered, was evaluated on the clinical test set. The predicted ROIs and LS/GLS were compared with the human annotated ground truth.

### Performance metrics

Model performance was primarily evaluated using the average absolute difference (AAD) between the GLS calculated from the DL-predicted ROI and the human annotated ROI (ground truth). AAD is defined as

$$AAD=\frac{{\left|{ GLS}_{DL}- { GLS}_{Clinician }\right|}_{patient\;1}+ \dots . {\left|{ GLS}_{DL}- { GLS}_{Clinician }\right|}_{patient\;n.}}{number\;of\;patients}.$$

GLS was calculated by averaging the longitudinal strain (LS) from all three apical views where present. Single-view LS was used to compare data from incomplete exams. DL obtained strain values were compared to clinical strain values on the basis of AAD with a 95% confidence interval (CI), and a Bland–Altman plot with a 95% limit of agreement (LOA) and relative bias was used to evaluate the distribution of the results. Note that strain is reported in percent and that the AAD is reported in percentage points.

When developing the model only standard performance metrics for segmentation, Dice score and Hausdorff distance (HD) were employed. These are metrics for geometrical overlap between the DL annotated area A_DL_ and the clinical annotated area A_Clinician,_ and their geometrical shape. The Dice score is defined as $D = 2 (|{ A}_{DL} \cap { A}_{Clinician }|) / (|{ A}_{DL} | + |{ A}_{Clinician}|)$. The coefficient is on a scale from 0 to 1, where 0 represents no overlap and 1 is a perfect overlap. The Hausdorff distance is a measure of the distance for each point on shape A to any point on shape B and is useful for measuring the similarity in shapes between two shapes.

The number of failures were defined as DL-predicted ROIs that were discontinuous or bifurcated, and/or included parts of the right ventricle, papillary muscle, or structures beyond the heart valves.

All statistical analyses were done using STATA SE 17.0 (Statacorp LLC, Texas, USA), Microsoft Excel version 2204 (Microsoft Corporation, Washington, USA) and Python 3.7.

## Results

**Fig. 3:**
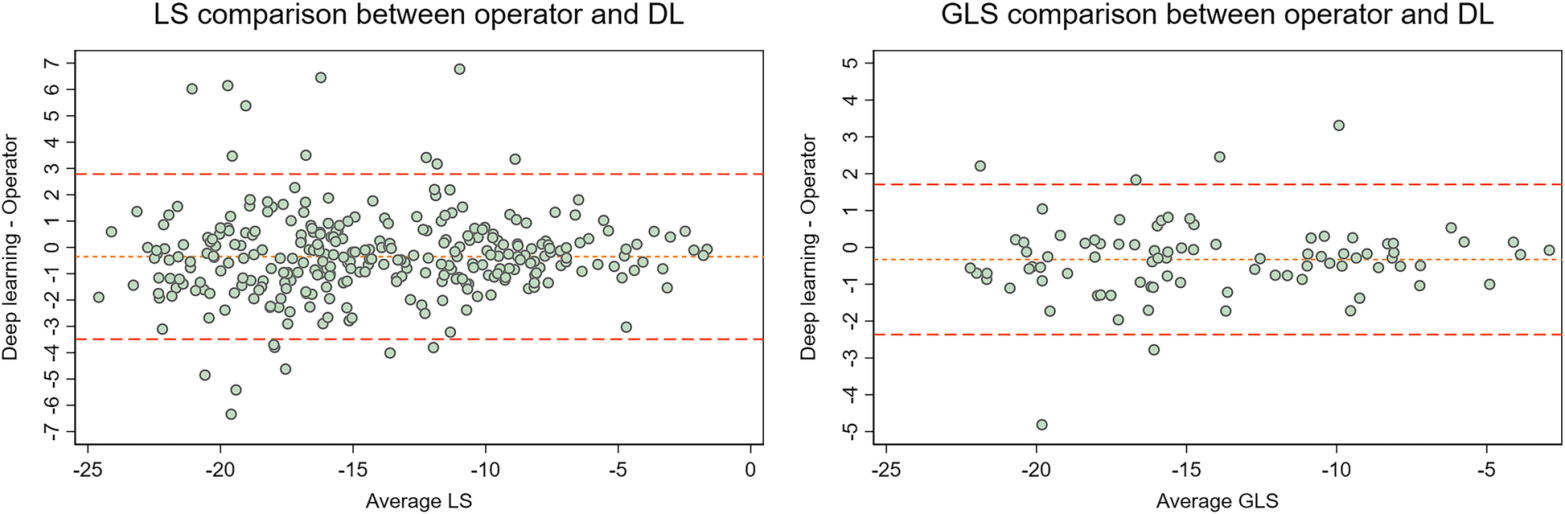
Bland–Altman plot for comparison of DL and Operator values for LS and GLS for best performing model The 95% limit of agreement were -2.366 – 1.708 with a relative bias of -0.329 for GLS, and -3.489 – 2.785 with a relative bias of -0.352 for LS. LS = single projection longitudinal strain, GLS = Global longitudinal strain, DL = Deep learning

Considering both GLS and LS results, the best overall model architecture performance was an EfficientNetB1 model pretrained on the CAMUS dataset and then trained on all data in the current dataset, having a AAD of 0.75 (95% CI 0.58–0.92) for GLS, and a AAD of 1.16 (95% CI 1.03–1.29) for LS. A Bland–Altman plot (Fig. 3) revealed no obvious bias, though there were fewer outliers where the average LS values were low.

### Impact of size and quality of training data

Evaluating the effect of cohort size on model performance (Table 2), Dice, HD and LS all show a non-significant trend towards improvement with increasing training set size, with a less prominent trend when the model was pretrained on CAMUS data. Limiting the training data set to only high and mid quality acquisitions did not improve model accuracy. Exams used in previous research produced models with better evaluation metrics than those trained on clinical exams only (Research dice 0.757 vs clinical dice 0.743).

**Table 2:** HD, DICE, LS and failures for data quality and model property testing

	**Geometric evaluation**		**Clinical measures evaluation**		**Number of failures**
	**Dice**	**HD**	**LS**	**GLS (95% CI)**	
**Cohort size for training**					
*n* = 100	0.739	14.51	1.46	0.97 (0.78–1.16)	13
*n* = 200	0.762	12.65	1.27	0.86 (0.69–1.02)	7
*n* = 300	0.762	12.02	1.23	0.79 (0.61–0.97)	3
*n* = 400	0.770	12.40	1.25	0.82 (0.64–1.00)	2
***n*** **= 518**^**a**^	0.779	11.52	1.17	**0.74 (0.59–0.90)**	3
**Cohort size for training w/CAMUS transfer learning**					
*n* = 100	0.759	12.56	1.28	0.81 (0.65–0.98)	9
*n* = 200	0.767	12.14	1.20	0.81 (0.61–0.92)	7
*n* = 300	0.767	11.83	1.21	0.75 (0.58–0.93)	4
*n* = 400	0.776	11.55	1.26	0.87 (0.69–1.04)	3
*n* = 518	0.779	**11.17**	**1.16**	0.75 (0.58–0.92)	2
**Exam quality**					
**H/M/L**^**a**^	0.779	11.52	1.17	**0.74 (0.59–0.90)**	**3**
H/M	0.769	12.41	1.23	0.81 (0.65–0.97)	2
**Image acquisition setting**					
From research (*n* = 219)	0.757	12.69	1.29	0.82 (0.62–1.02)	3
From clinical exams (*n* = 219)	0.743	13.94	1.32	0.85 (0.67–1.03)	8
**Transfer learning**					
ImageNet	**0.786**	11.43	1.22	0.76 (0.58–0.92)	2
CAMUS	0.779	**11.17**	**1.16**	0.75 (0.58–0.92)	2
**CAMUS set as training set**					
CAMUS baseline	0.682	21.74	1.80	1.65 (1.35–1.95)	17

The same baseline Efficientnet model with augmentation and HML were employed for all tests. Where not otherwise stated all data was used

*HD* Hausdorff distance, *LS* Single projection longitudinal strain, *GLS* Global longitudinal strain, *CI* Confidence interval

^a^Reference model

### Transfer learning

Two different datasets were used for pre-training the CNN in order to perform the transfer learning experiments. The model pretrained on ImageNet and further trained on the dataset proposed in this study improved the performance in terms of Dice score vs the reference model only trained on the proposed dataset (all available data) (ImageNet Dice 0.786 vs reference Dice 0.779), but with diverging results on HD and LS. However, the differences between the pretrained model and the reference model were not statistically significant.

### Network architecture

Finally, evaluating encoder differences between standard U-net, ResNet50 and EfficientNet (Table 3), again the different performance metrics gave disparate results. The traditional U-net and the newer EfficientNet B1 gave the best results for LS (1.16 percentage points), ResNet50 the best Dice score, and EfficientNet B1 the best GLS and HD results.

**Table 3:** Comparison of model architecture based on AAD for LS and GLS with 95% CI

	**Dice**	**HD**	**LS (95% CI)**	**GLS (95% CI)**
EfficientNetB1	0.779	**11.17**	**1.16** (1.03–1.29)	**0.75** (0.58–0.92)
U-net	0.774	12.12	**1.16** (1.03–1.28)	0.77 (0.61–0.94)
ResNet50	**0.783**	11.62	1.18 (1.05–1.31)	0.78 (0.62–0.94)

Models were trained on High, medium and low quality data, and utilized augmentations and CAMUS transfer learning

*HD* Hausdorff distance, *LS* Single projection longitudinal strain, *GLS* Global longitudinal strain, *CI* Confidence interval

### Result summary

The numerical differences between cohort compositions and technological approaches to model training were minor in terms of geometric comparisons HD and Dice as shown in both Tables 2 and 3. With regards to the clinical output parameters LS and GLS, the variation between models was greater. However, except for the model trained on CAMUS data, GLS was within 0.74–0.81 percentage points and LS within 1.16–1.32 percentage points of ground truth for all models.

### Model failure and results outliers

The number of failures for the highest performing models were 2 or 3, giving a failure rate of less than 1%. There was a higher failure rate in the models trained on smaller cohort size, but the effect was diminishing after a cohort size of 300. The model trained on the external dataset, CAMUS, had the highest failure rate of 5.54% (17 out of 307).

**Fig. 4:**
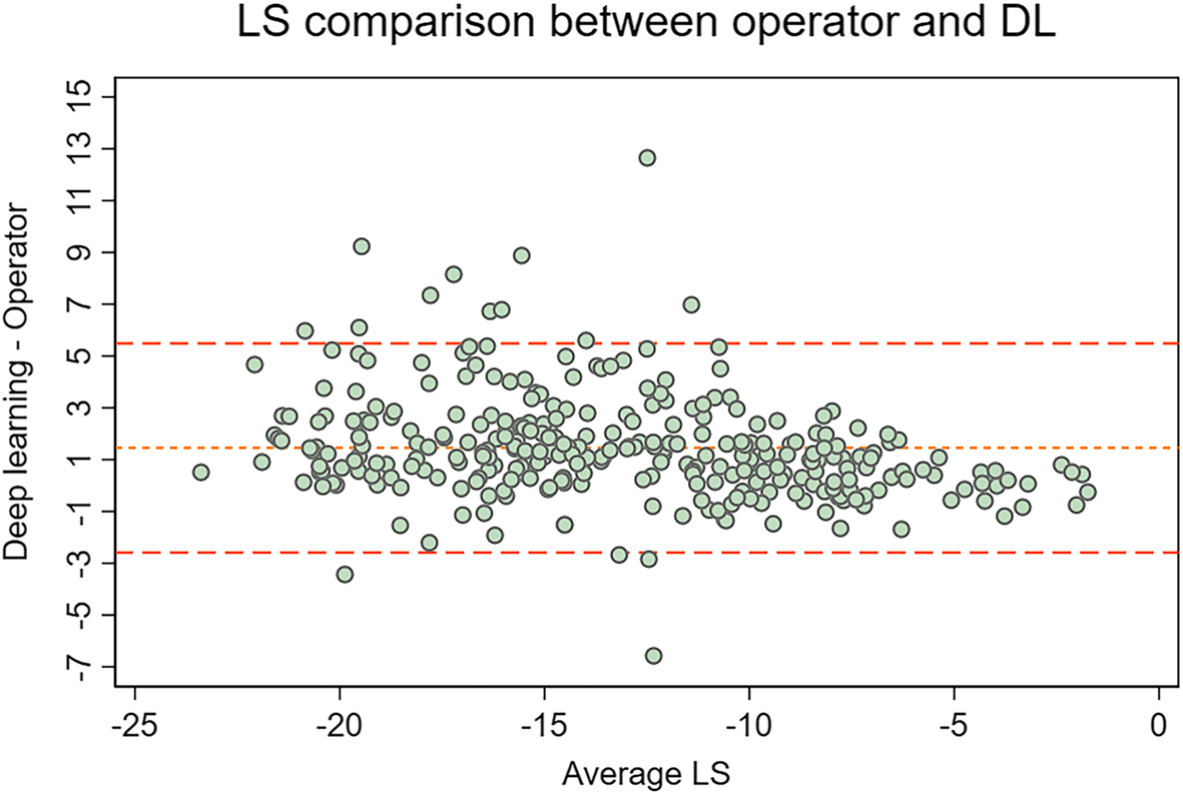
Bland–Altman plot for comparison of DL trained on CAMUS and operator values The 95% limits of agreement were -1.51 – 4.53 with a relative bias of 1.51 for GLS, and -2.58 – 5.49 with a relative bias of 1.45 for LS. LS = Single projection longitudinal strain, DL = Deep learning

A significant outlier in performance came from the model trained on the CAMUS dataset. The model had a AAD in GLS of 1.65 (95% CI 1.35–1.95) and AAD in LS of 1.80 (95% CI 1.60–2.00). A Bland–Altman plot (Fig. 4) revealed a trend of the DL overestimating the GLS and LS.

## Discussion

Our results demonstrate that deep learning can automate strain calculations with comparable accuracy to trained cardiologists. Considering that the fully automated strain calculations were within interobserver variation observed in other studies [12, 28], the difference is within a range commonly accepted between operators. It is worth mentioning that DL models such as those utilized in this study have no intraobserver variation [12]. To the authors’ knowledge, at the time of writing, this is currently the largest dataset employed for DL automated strain calculations, and the only paper that explores the combination of an in-house developed DL model and commercial echocardiographic analysis software to calculate LS/GLS. Our approach achieved a smaller 95% LOA and bias in GLS than what was reported by Salte et al. [12], and a smaller absolute deviation in GLS compared to Zhang et al. [13]. Moreover, our approach achieved a smaller absolute error in strain estimation compared to DL predicted circumferential and radial strain on Magnetic Resonance Imaging [29].

### Effect of tested parameters and techniques

In the general DL image segmentation space, papers report on new network architectures improving geometric comparison scores such as Dice on reference datasets. Often, improvements from the previous state-of-the-art model are small, and at the third significant digit level. In our experiments, different DL approaches and clinical parameters were applied to real world, noisy echocardiographic data, and the incremental gains from presumed improved model building approaches were minimal.

### Training data quality

Although differences between models were small, some outcomes are worth noting. Previous studies have removed low quality images from training sets. It was therefore noteworthy that including low quality data slightly increased model performance based on Dice score, HD, LS, and GLS, while only resulting in one more failure (Table 2). This indicates that the increase in training size and variation in quality might improve DL models—at least in DL settings where data set size is a limiting factor, as is often the case with medical imagery. However, when low quality images can lead to inaccurate annotation or labeling of training data, the value of inclusion of such images is still debatable.

### Image acquisition setting

The initial hypothesis was that exams conducted in a busy clinical practice would be less precise than research analysis, leading to a worse performing model when training on clinical data. The model trained on research data had a lower failure rate, indicating that it is more stable. However, image acquisition setting did not appear to impact model performance in terms of LS and GLS (Table 2). The results can indicate there are more outliers in terms of image and ROI quality in clinical data, resulting in more failures, but that models trained on such data still can become accurate overall. It should also be noted that increasing training size lowered failure rate, which is encouraging considering clinical data is generally more available.

### Training set size

As stated earlier, limited data availability is a challenge for DL in medical imaging. It is encouraging that increasing the patient number beyond 100–200 patients only has a limited effect on model performance (Table 2). These findings correspond well with the findings of Leclerc et al. [20], and shows the potential of utilizing DL even where there is limited available data. It is important to note that the higher failure rate when training on smaller cohort sizes can cause poor exams to be eliminated, slightly elevating the perceived model performance for these models. Again, the numerical variation is slight, and the signal-to-noise ratio of echocardiographic images may be the most important limiting factor.

### Transfer learning

Transfer learning seemed to decrease the number of patients needed to achieve acceptable results. However, there are indications that transfer learning benefits vary depending on the dataset size and complexity [30]. The diverging results when comparing ImageNet and The CAMUS dataset supports this finding. Finally, it is interesting that the relatively old U-net network architecture performed just as well as the newer networks (Table 3), considering the large improvement to neural networks and their performance in other fields. The challenges proposed by the low resolution, low contrast echocardiographic images for segmentation are widely recognized, and the results indicate that the quality of training and ground truth data is still the biggest challenge to improve DL for strain analysis.

### Training on CAMUS

The model that was trained on the CAMUS dataset (Fig. 4) was an outlier in terms of performance, and it generally overestimated strain compared to expert operators. In a visual comparison of the ROIs, the CAMUS ROIs tended to be larger than the operator outlined ones in our dataset. The overestimation can be a result of differences in operator tendencies between the originating hospitals, and highlights the importance of DL model validation on local data before implementation both in research and clinical practice.

### Clinical significance

Since our approach follows the clinical workflow step by step, the operator can monitor and correct any mistakes of the DL segmentation, thereby retaining the quality and accountability of the analysis. Automating strain calculations has the potential to provide both higher quality patient care by increasing the availability of strain analysis, as well as providing more time for patients by reducing time spent per analysis. Utilizing such models will also allow for the analysis of enormous amounts of databases for research, making later studies more efficient and less costly.

### Limitations

Our study contains a diverse set of heart diseases, annotated by a variety of operators. The heterogeneity of the dataset should produce a more generalizable DL model. However, the model has yet to be externally validated on data from other populations, other hospitals, and data sets achieved from other echocardiographic machines. Some major heart diseases, such as atrial fibrillation, are not represented in the study due to lack of availability. While it is reportedly more difficult to calculate strain from patients with atrial fibrillation, our approach should not be more affected than clinical practice as it follows the clinical workflow. The proposed approach would most likely still be able to increase efficiency and reproducibility in these patients. Furthermore, image segmentation using DL models is a rapidly evolving field. During the course of this study, newer architectures have emerged that demonstrate a higher performance on the benchmarking dataset, ImageNet, compared to those employed in the current study.

## Conclusion

Our study, which was performed before automated strain analysis became available in commercial software, confirms that automatic strain calculations are feasible and that the results are within a range of variations that are appropriate for echocardiographic experts. This study further confirms that automatic LS measurements using a DL model could be integrated into readily available commercial echocardiographic analysis software, but also demonstrates the risk of bias in datasets used for model training.

## Data Availability

The datasets utilized during the current study were deemed by the local data protection officer to contain patient sensitive data that prohibits it from publication. Anonymized results and CNN code are available from the corresponding author on reasonable requests.

## Abbreviations

AAD: Average absolute difference
AI: Artificial intelligence
ASE: The American Society of Echocardiography
CI: Confidence interval
CNNs: Convolutional neural networks
DL: Deep learning
EACVI: European Association of Cardiovascular Imaging
GLS: Global longitudinal strain
HD: Hausdorff distance
LOA: Limit of agreement
LS: Longitudinal strain
LV: Left ventricle
ROI: Region of interest
STE: Speckle tracking echocardiography
